# Supplementary figures and images for: An osteocalcin-deficient mouse strain without endocrine abnormalities
Source: PLoS Genet. 2020 May 28;16(5):e1008361. doi: 10.1371/journal.pgen.1008361 (PMC7255615; doi:10.1371/journal.pgen.1008361)

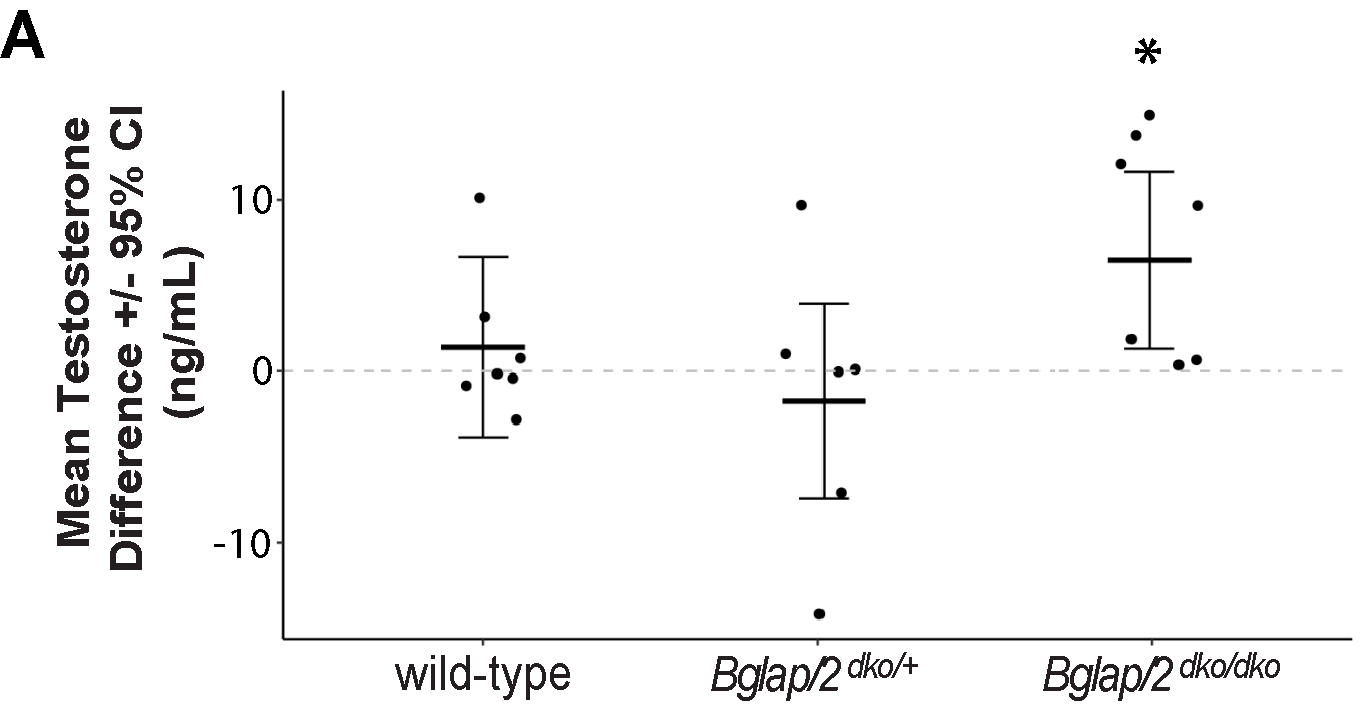

Supplement: S1 Fig — The mean difference of the change in testosterone is displayed using a +/- 95% confidence interval (CI). The difference in testosterone was calculated by subtracting the day 0 value from the day 3 value (* represents p > 0.05). (TIF) [file pgen.1008361.s001.tif]
