# Supplementary material for: An osteocalcin-deficient mouse strain without endocrine abnormalities
Source: PLoS Genet. 2020 May 28;16(5):e1008361. doi: 10.1371/journal.pgen.1008361 (PMC7255615; doi:10.1371/journal.pgen.1008361)
Supplement: S1 Table — Individual measurements for each mouse are shown for ultimate force, stiffness, and energy to FU. Bglap/2dko/dko (KO/KO, n = 12) and wild-type (WT, n = 12) are noted, and the average and standard deviation are presented for each variable. (DOCX) [file pgen.1008361.s002.docx]

| Animal ID | Genotype | | Ultimate Force (N) | | Stiffness (N/mm) | | Energy to FU (mJ) |
| --- | --- | --- | --- | --- | --- | --- | --- |
| 39763 | WT | 18.70 | | 87.40 | | 3.997 | |
| 42022 | WT | 23.00 | | 100.82 | | 4.154 | |
| 42027 | WT | 19.25 | | 106.67 | | 2.834 | |
| 42185 | WT | 17.50 | | 48.12 | | 4.301 | |
| 42607 | WT | 19.25 | | 111.58 | | 3.048 | |
| 42613 | WT | 24.95 | | 106.01 | | 5.520 | |
| 42616 | WT | 24.05 | | 139.29 | | 5.029 | |
| 42622 | WT | 17.00 | | 82.27 | | 3.180 | |
| 42628 | WT | 23.80 | | 119.23 | | 3.418 | |
| 43180 | WT | 23.00 | | 124.98 | | 4.384 | |
| 43784 | WT | 21.85 | | 122.96 | | 4.064 | |
| 43785 | WT | 17.60 | | 115.44 | | 2.417 | |
| 39762 | KO/KO | 21.75 | | 128.16 | | 3.528 | |
| 42020 | KO/KO | 21.80 | | 120.42 | | 3.673 | |
| 42021 | KO/KO | 18.45 | | 117.55 | | 3.706 | |
| 42026 | KO/KO | 17.55 | | 97.43 | | 2.815 | |
| 42028 | KO/KO | 18.05 | | 100.01 | | 3.579 | |
| 42184 | KO/KO | 20.75 | | 90.82 | | 4.794 | |
| 42623 | KO/KO | 18.90 | | 111.94 | | 3.332 | |
| 42626 | KO/KO | 29.00 | | 121.57 | | 6.486 | |
| 42629 | KO/KO | 24.25 | | 123.93 | | 3.909 | |
| 43181 | KO/KO | 24.25 | | 139.15 | | 4.483 | |
| 43799 | KO/KO | 20.20 | | 112.56 | | 3.336 | |
| 43800 | KO/KO | 23.25 | | 130.37 | | 4.612 | |
|  |  |  | |  | |  | |
| Genotype | **Index** | | **Ultimate Force (N)** | | **Stiffness (N/mm)** | | **Energy to FU (mJ)** |
| WT | Average | 20.83 | | 105.40 | | 3.86 | |
|  | Standard Deviation | 2.90 | | 24.00 | | 0.91 | |
| KO | Average | 21.52 | | 116.16 | | 4.02 | |
|  | Standard Deviation | 3.31 | | 14.36 | | 0.97 | |
