# Supplementary material for: An osteocalcin-deficient mouse strain without endocrine abnormalities
Source: PLoS Genet. 2020 May 28;16(5):e1008361. doi: 10.1371/journal.pgen.1008361 (PMC7255615; doi:10.1371/journal.pgen.1008361)
Supplement: S2 Table — Individual measurements for each mouse are shown for ultimate force, stiffness, and energy to FU. Bglap/2dko/dko (KO/KO, n = 14) and wild-type (WT, n = 18) are noted, and the average and standard deviation are presented for each variable. (DOCX) [file pgen.1008361.s003.docx]

| Animal ID | Genotype | Ultimate Force (N) | Stiffness (N/mm) | Energy to FU (mJ) |
| --- | --- | --- | --- | --- |
| 39773 | WT | 20.45 | 106.94 | 3.483 |
| 39853 | WT | 20.95 | 88.69 | 3.992 |
| 40062 | WT | 21.95 | 106.20 | 4.558 |
| 40471 | WT | 23.85 | 117.17 | 4.868 |
| 40473 | WT | 22.65 | 91.44 | 4.426 |
| 40481 | WT | 23.65 | 141.13 | 4.218 |
| 40482 | WT | 24.90 | 120.46 | 3.851 |
| 40483 | WT | 20.95 | 109.02 | 3.217 |
| 42187 | WT | 17.45 | 97.09 | 2.969 |
| 42188 | WT | 21.80 | 79.58 | 5.146 |
| 42609 | WT | 22.55 | 75.32 | 6.272 |
| 42612 | WT | 22.35 | 97.20 | 3.811 |
| 42632 | WT | 25.70 | 138.12 | 4.968 |
| 42633 | WT | 21.45 | 116.35 | 4.767 |
| 42635 | WT | 22.55 | 113.04 | 5.267 |
| 42636 | WT | 24.10 | 124.95 | 4.884 |
| 43189 | WT | 23.35 | 127.40 | 4.704 |
| 43804 | WT | 25.90 | 137.80 | 4.677 |
| 39771 | KO/KO | 17.15 | 74.61 | 2.823 |
| 39854 | KO/KO | 23.50 | 111.92 | 5.701 |
| 39857 | KO/KO | 19.55 | 118.96 | 2.512 |
| 40063 | KO/KO | 17.95 | 86.87 | 2.408 |
| 40475 | KO/KO | 19.10 | 89.83 | 3.718 |
| 40486 | KO/KO | 18.75 | 92.37 | 3.051 |
| 42189 | KO/KO | 20.05 | 87.63 | 4.069 |
| 42608 | KO/KO | 22.25 | 136.22 | 4.899 |
| 42611 | KO/KO | 21.15 | 105.36 | 4.010 |
| 42634 | KO/KO | 23.05 | 144.27 | 4.420 |
| 43178 | KO/KO | 17.30 | 100.36 | 2.154 |
| 43186 | KO/KO | 20.70 | 112.32 | 4.940 |
| 43793 | KO/KO | 24.00 | 141.34 | 4.859 |
| 43803 | KO/KO | 22.35 | 132.34 | 4.194 |
|  |  |  |  |  |
| Genotype | **Index** | **Ultimate Force (N)** | **Stiffness (N/mm)** | **Energy to FU (mJ)** |
| WT | Average | 22.59 | 110.44 | 4.45 |
|  | Standard Deviation | 2.03 | 19.62 | 0.80 |
| KO | Average | 20.49 | 109.60 | 3.84 |
|  | Standard Deviation | 2.30 | 22.45 | 1.10 |
