# Supplementary material for: An osteocalcin-deficient mouse strain without endocrine abnormalities
Source: PLoS Genet. 2020 May 28;16(5):e1008361. doi: 10.1371/journal.pgen.1008361 (PMC7255615; doi:10.1371/journal.pgen.1008361)
Supplement: S3 Table — Quantitation of variables for Bglap/2dko/dko (KO/KO, n = 4 or 3) and wild-type (n = 3) female mice are shown. (DOCX) [file pgen.1008361.s004.docx]

**FTIR Imaging Results for Cortical Bone**

| Sample | Mineral/matrix | Carbonate/mineral | Collagen crosslink maturity | Crystallinity | Acid Phosphate content (HPO_4_^2-^) |
| --- | --- | --- | --- | --- | --- |
| Wild-type  (n=3) | 9.51 +/- 0.35 | 0.0075 +/- 0.0002 | 3.87 +/- 0.06 | 1.2 +/- 0.01 | 0.42 +/- 0.01 |
| *Bglap/2^dko/dko^* (n=4) | 8.86 +/- 0.29 | 0.0082 +/- 0.00008 | 4.28 +/- 0.03 | 1.18 +/- 0.01 | 0.41 +/- 0.01 |
| significance | n.s. | p < 0.01 | p < 0.01 | n.s | n.s. |

**FTIR Imaging Results for Trabecular Bone**

| Sample | | Mineral/matrix | Carbonate/mineral | Collagen crosslink maturity | Crystallinity | Acid Phosphate content (HPO_4_^2-^) |
| --- | --- | --- | --- | --- | --- | --- |
| Wild-type (n=3) | 5.3 +/- 0.10 | | 0.0057 +/- 0.0002 | 4.79 +/- 0.13 | 1.2 +/- 0.02 | 0.66 +/-0.01 |
| *Bglap/2^dko/dko^* (n=3) | 5.4 +/- 0.22 | | 0.006 +/- 0.0003 | 5.11 +/- 0.25 | 1.1 +/- 0.02 | 0.62 +/- 0.02 |
| significance | n.s. | | n.s. | n.s. | n.s. | n.s. |
