# Supplementary material for: An osteocalcin-deficient mouse strain without endocrine abnormalities
Source: PLoS Genet. 2020 May 28;16(5):e1008361. doi: 10.1371/journal.pgen.1008361 (PMC7255615; doi:10.1371/journal.pgen.1008361)
Supplement: Attachment — Submitted filename: PLOS Genetics Reply 022020.docx [file pgen.1008361.s006.docx]

February 14, 2020

RESPONSE TO REVIEWER AND EDITOR COMMENTS RE: PGENETICS-D-19-01327

We were pleased by the overall positive nature of the reviewers’ comments regarding our manuscript. We have made the following revisions to the manuscript based on our correspondence with the editors. In addition, we have also included a separate version of the manuscript text in which any area in which substantial revisions have taken place is marked in red text within yellow highlights.

**Editorial Request 1. Specifically, we ask that you make a clear acknowledgement of the limitations of your current data to detect subtle alterations in glucose sensing and insulin secretion and include appropriate discussion around this important point.**

We have added text in the discussion that clearly acknowledges the limitations of the current glucose metabolism assessment and also explicitly acknowledges that more detailed analysis of these mice may uncover more subtle phenotypes. To be fair, we also added a similar acknowledgement about fertility-related phenotypes. In both cases, we have encouraged interested investigators to obtain these mice from the Jackson Laboratories repository to carry out independent, detailed analyses.

**Editorial Request 2. We also ask that you include that data from the additional mice you now have available to address the second major point raised around testosterone levels. You should also include the additional technical information on the timing of testosterone measurements and other information to confirm that stringent efforts were made to avoid unnecessary technical variations.**

We have performed testosterone measurements on an independent cohort of mice. In this case, measurements were performed on samples we shipped to the NIH P30-supported University of Virginia Ligand Assay & Analysis Core of the Center for Research in Reproduction for analysis (the core personnel were blinded to genotypic identity). We provide extensive detail on how these mice were handled prior to the sample collection (singly housed, virgin males) and provide specific information the caging systems used in our animal rooms. We also provide additional context into how the wide variation we see in testosterone measurements (even in the same animal 3 days apart) is consistent with previous studies. This is due to mouse hepatocytes producing little sex hormone binding globulin (SHBG), the principal carrier of testosterone in blood, in contrast to the abundant production of SHBG in human liver.

**Editorial Request 3. Finally, we request you include additional an discussion about the potential role of the osteocalcin fragment.**

We have provided additional discussion to clarify that the that the first 23 amino acids of the putative 25 amino acid OCN fragment we described is composed of the signal peptide and therefore only a 2 amino acid fragment would be left after signal peptidase cleavage.

We also provided an updated image for Figure 3 and updated Supplemental Table 3 (the FTIR analysis). This did not change any conclusions of the study.

We thank the reviewers and editors for the opportunity to submit this revised manuscript and are happy to provide additional information if necessary.

Sincerely,

Bart Williams (on behalf of Matt Warman and Alex Robling)

January 24, 2020

Editorial Response to December 6, 2019 Queries

Dear Dr. Williams,

Thank you for your patience while the editors assessed your request. I now have the following response for you:

"Dear Dr Williams,

We have carefully reviewed your letter responding to the reviewers’ comments and we are willing to consider a revised manuscript generally along the lines you propose.

Specifically, we ask that you make a clear acknowledgement of the limitations of your current data to detect subtle alterations in glucose sensing and insulin secretion and include appropriate discussion around this important point.

We also ask that you include that data from the additional mice you now have available to address the second major point raised around testosterone levels. You should also include the additional technical information on the timing of testosterone measurements and other information to confirm that stringent efforts were made to avoid unnecessary technical variations.

Finally, we request you include additional an discussion about the potential role of the osteocalcin fragment.

We hope that you are able to make these changes in a timely way and look forward to receiving your revised manuscript in the next few weeks, with a proposed deadline of 15th February."

Please don't hesitate to get in touch if you have any questions.

Kind regards,
Alice

December 6, 2019

RESPONSE TO REVIEWER COMMENTS RE: PGENETICS-D-19-01327

We were pleased by the overall positive nature of the reviewers’ comments regarding our manuscript. We are emailing with an update of the status of revisions to the manuscript and in the hope of receiving guidance over what the editors and reviewers would consider an appropriate response to facilitate acceptance.

**Response to Reviewer #1.**

We thank the reviewer for their strong supportive comments and will spell out FPKM suggested.

**Response to Reviewer #2**

We thank the reviewer for their strongly support of the value of our studies. This review provided three comments to address that they felt would enhance the manuscript. These three points are listed below.

1. **“….challenge the mice with a glucose tolerance test to see if that brings out a difference in glucose metabolism missed by the studies that were performed.”**

As presented in the initial version of the manuscript, we carried out analysis of glucose levels in random fed and fasted animals and saw no detectable differences between control littermates and mutant animals. Because we were not actively maintaining cohorts of these animals, we have had to build up the numbers of animals in our colonies to have the proper number of animals to properly power these studies. We estimate that it would take 2-3 additional months to have sufficient animals at the proper age to carry out this study.

One alternative we would respectfully propose would be to write a discussion into the manuscript that emphasizes that we have not carried out experiments such as glucose tolerance tests and that we hope that the availability of these animals in a public repository will enable numerous laboratories to obtain them and carry out very detailed studies to very thoroughly evaluate this question.

2. **Testis weight and volume measurements involved only four WT mice. The weights and volumes of the KOs were lower, but apparently not statistically so. Particularly given the wide variation in the WT testis weight (20-30%), the authors need to do a power calculation to determine how many mice need to be studied to avoid missing a real difference, and repeat this study with adequate power. Testosterone levels vary substantially in male mice, depending on whether they are being housed with females or not. No mention was made of precautions to minimize variation among the mice. Was testosterone measurement made at the same time of day to minimize variation due to diurnal variation? When the findings are that the mice are not different, then substantial efforts should be made to avoid missing real differences.**

The males used to assess the testosterone levels were virgin males who were singly housed for at least 7 days prior to sample collection.

The reviewer accurately points out that testosterone levels vary widely in mice, and we did attempt to minimize variation as stated above. Our expansion of the colonies in the last two month likely provides us with additional males (controls and mutants) that could be further analyzed for testosterone levels to add additional information to this portion of the report. We would mention that the levels of Testosterone observed by the Karsenty group were well below the levels seen in our mice (either wild type or controls).

**3. The nature of the deletion in the KO mouse appears to allow the possible expression of a peptide fragment of osteocalcin. How do the authors know that this isn’t sufficient to rescue the pancreatic and muscle effects of osteocalcin? This seems unlikely to me, but needs to be discussed more fully by the authors. Has there been any structure-function analysis of these proposed actions of osteocalcin?**

We will provide a more detailed discussion of potential role of the remaining fragment of OCN having some signaling activity.

that we perform a glucose tolerance test to compare the response of Osteocalcin-deficient mice to their littermates in that context. The Karsenty Laboratory have previously reported that there is a significant difference in their Ocn-mutant mice relative to wild type control mice in this context. The current status of our mouse colony will not allow these studies to be performed at the 3-month-old timepoint used by Karsenty and colleague for another 5-6 weeks.

**Response to Reviewer 3**

This reviewer was also strongly supportive and made 4 minor comments that will address within the document to further clarify the content.

Thank you for your consideration.

Sincerely,

Bart Williams
